# GBS-Based Deconvolution of the Surviving North American Collection of Cold-Hardy Kiwifruit (*Actinidia* spp.) Germplasm

**DOI:** 10.1371/journal.pone.0170580

**Published:** 2017-01-26

**Authors:** Arthur T. O. Melo, Robert S. Guthrie, Iago Hale

**Affiliations:** 1 University of New Hampshire, College of Life Sciences and Agriculture, Department of Biological Sciences, Durham, New Hampshire, United States of America; 2 Minnesota Landscape Arboretum Horticultural Research Center, University of Minnesota, Chanhassen, Minnesota, United States of America; Wuhan Botanical Garden, CHINA

## Abstract

Plant germplasm collections can be invaluable resources to plant breeders, provided they are well-characterized. After 140 years of acquisition and curation efforts by a wide and largely non-coordinated array of private and institutional actors, the current US collection of cold-hardy kiwifruit (*Actinidia* spp.) is rife with misclassifications, misnomers, and mix-ups. To facilitate the systematic improvement and resource-efficient curation of these species of long-recognized horticultural potential, we used genotyping-by-sequencing (GBS) data to deconvolute this historic collection. Evaluation of a total of 138 accessions (103 *A*. *arguta*, 28 *A*. *kolomikta*, and 7 *A*. *polygama*) with an interspecific set of 1,040 high-quality SNPs resulted in clear resolution of the three species. Intraspecific analysis (2,964 SNPs) within *A*. *arguta* revealed a significant level of redundancy (41.7%; only 60 unique genotypes out of 103 analyzed) and a sub-population structure reflecting likely geographic provenance, phenotypic classes, and hybrid pedigree. For *A*. *kolomikta* (3,425 SNPs), the level of accession redundancy was even higher (53.6%; 13 unique genotypes out of 28 analyzed); but no sub-structure was detected. Numerous instances were discovered of distinct genotypes sharing a common name, different names assigned to the same genotype, mistaken species assignments, and incorrect gender records, all critical information for both breeders and curators. In terms of method, this study demonstrates the practical and cost-effective use of GBS data to characterize plant genetic resources, despite ploidy differences and the lack of reference genomes. With the recent prohibition on further imports of *Actinidia* plant material into the country and with the active eradication of historic vines looming, this analysis of the US cold-hardy kiwifruit germplasm collection provides a timely assessment of the genetic resource base of an emerging, high-value specialty crop.

## Introduction

With its first commercial planting in the early 1930's, the fuzzy kiwifruit is arguably the most recently domesticated agricultural plant species of global significance [1]. While the nearly $2.5 billion (USD) international kiwifruit industry today [2] is based almost entirely on two temperate species, *Actinidia chinensis* var. *deliciosa* and *A*. *chinensis* var. *chinensis*, other cold-hardier species within the highly diverse *Actinidia* genus have long been recognized for their horticultural potential, particularly in more northern latitudes [3]. Foremost among these are *A*. *arguta* and *A*. *kolomikta*, species of negligible commercial production [4], though ones with an extensive history of use by landscape architects, gardeners, and novel fruit enthusiasts in the United States since the first introduction of *A*. *arguta* seeds from Japan by Massachusetts Agricultural College President William S. Clark in the fall of 1877 (S1 Fig).

Table grape-sized with a palatable, hairless skin, an impressive nutritional profile [5], and a pleasingly complex sweet-acid flavor [6], the berry-like fruits of these species, particularly *A*. *arguta*, are coalescing in the marketplace under the name "kiwiberries", a variant of a term initially used to refer to fruits of *A*. *chinensis* var. *deliciosa* [7]. Interest in domesticating and developing the commercial potential of these Asiatic species continues to grow, as evidenced by renewed USDA investment in their improvement and the gradual increase in planted acreage in the Northeast, mid-Atlantic, Great Lakes, and Pacific Northwest regions of the United States over the past 5 years (D. Jackson, K. Demchak, R. Ort, R. Guthrie, and M. Hurst, pers. comms.). One possible driver of this trend may be the increasing demand for novel, high-value, seasonal, and locally-grown produce by consumers in those regions [8, 9]. Such a market shift certainly favors the kiwiberry, a fruit whose small size and relatively short post-harvest life were once viewed as impediments to its commercial success [4].

While commercial interest in kiwiberries is rising, the germplasm available to support systematic improvement efforts in the United States is on the decline. For example, of the 308 recognizable records of *A*. *arguta*, *A*. *arguta* hybrid, *A*. *kolomikta*, and *A*. *kolomikta* hybrid accessions in the USDA National Plant Germplasm System (NPGS) database, 163 (52.9%) are inactive (i.e. no longer held within the NPGS) and 134 (43.5%) are currently unavailable, leaving only 11 (3.6%) available for public distribution [10]. A primary reason for this dramatic reduction is likely economic: Cold-hardy *Actinidia* vines are vigorous perennial lianas that require substantial infrastructure (e.g. trellises, pergolas, etc.), manual training, multiple prunings annually, and a sizeable land base (15–20 m^2^ per mature vine in commercial operations), making their curation a highly resource-intensive endeavor.

Outside the nursery trade and the NPGS, extant diversity is also eroding due to the ongoing decline of historic landscape commissions from the period spanning the 1880’s to the 1930’s, a time when these species were widely used as ornamental "utility class” vines on private estates, botanical gardens, and institutions throughout the Northeast and mid-Atlantic regions [11]. More recently, such extirpation has been exacerbated by efforts to intentionally eradicate *A*. *arguta* from certain regions, due to claims of invasiveness [12, 13] of vines that are actually abandoned remnants of historically-documented plantings. Finally, the germplasm available to kiwiberry improvement programs in the United States was further restricted in November 2010 by a permanent ban on the importation of any live *Actinidia* plant material to prevent the introduction of the bacterial pathogen *Pseudomonas syringae pv*. *actinidiae* (Psa) into the country [14].

Compounding the problem of germplasm loss is the fact that the genetic diversity of the surviving collection may be over-estimated. Indeed, among the accessions surviving today, significant convolution (i.e. redundancy, mis-characterization, and identity error) is likely, due to the long and complicated history of cold-hardy *Actinidia* spp. acquisition, curation, and dissemination in the United States. Since 1898, when the USDA established the Plant Exploration Program (PEP) to obtain plant material of potential economic value, various USDA-sponsored expeditions have acquired kiwiberry germplasm from different parts of eastern Asia. While many of the later acquisitions have remained within the domain of the NPGS since their collection, such is not the case with the earlier (pre-1980's) acquisitions (S1 Fig).

With the 1975 closure of the Chico Plant Introduction Station in Chico, CA, the initial era of formal USDA curation and selection of kiwiberry germplasm came to an end. By that time, much of the original *Actinidia* plant material no longer existed at the facility [15]. Of those accessions which remained, some were moved by S. Dietz to a clonal genebank at the Central Ferry location of the Western Regional Plant Introduction Station (W-6), while others were retrieved by commercial entities [e.g. Stanley & Sons, M. McConkey (Edible Landscaping), and others], thereby entering the nursery trade [16].

Cold-hardy *Actinidia* spp. once again came under the auspices of official USDA curation efforts with the establishment of the *Actinidia* collection at the Northwest Plant Germplasm Repository in Corvallis, OR, in 1981. In 1986, the mandate for curating this collection shifted to the National Clonal Germplasm Repository (NCGR) in Davis, CA, a more stressful environment for the cold-hardy species, only to return to Corvallis in 1999, then back to Davis in 2011. Since 1999 (K. Hummer, pers. comm.), the USDA has gradually regained some of the diversity previously forfeited to the nursery trade; but there are indications that those interim years were not without mix-ups. For example, in the description of cv. 'Dumbarton Oaks' offered by the nursery One Green World, the berries are described as fascinated, like small pumpkins, a description at odds with the smooth, oblong berries of cv. 'Dumbarton Oaks' offered by Edible Landscaping, PI 617135 currently in the USDA collection, and those of the source vine in Washington, DC. In another instance, cv. '127–40' has re-entered the USDA collections from two separate sources; but while one accession is male (PI617163), the other is female (PI617142). Another gender switch occurred with cv. 'Turrets,' a female selection originally offered by Teltane Farm & Nursery that now exists within the NPGS as a male accession.

Confusion regarding misnomers and usurpers among cold-hardy *Actinidia* accessions is nothing new; in fact, Clark's initial *A*. *arguta* collections from Japan were wrongly classified as *A*. *polygama*. But perhaps the best historic example of cultivar confusion is *A*. *arguta* cv. 'Ananasnaja' (a.k.a. 'Ananasnaya' and 'Anna'), the current mainstay of kiwiberry production in Oregon. The original 'Ananasnaja' (Актинидия Ананасая Мичурина) was derived from a third-generation *A*. *kolomikta* vine sown by Russian plant breeder I. Michurin in 1924 [17]. However, 14 years after Michurin’s death, the botanist V.A. Evreinoff described 'Ananas de Mitchourine' (Michurin’s Pineapple) as an interspecific *A*. *arguta* × *A*. *kolomikta* hybrid [18]. Subsequently, a genotype dubbed 'Ananasnaja' arrived in the USA in 1972 from Belgium as a full-fledged *A*. *arguta* cultivar [16]. Since 1981, the USDA has maintained no fewer than 14 different accessions in its repositories bearing some variant of the 'Ananasnaya' name, despite differences in gender and even species assignment.

To make reasonably efficient progress in developing improved cultivars of kiwiberry for commercial fruit production in light of declining genetic diversity, the inherent costliness of accession curation, and the likely convolution of available accessions, there is a clear need to assess what remains of the North American collection of cold-hardy *Actinidia* germplasm. The purpose of this study is to address this need. Using genotyping-by-sequencing (GBS) data to deconvolute the surviving lines in the USDA repository and the nursery trade, our intention is not only to enhance the resource efficiency of curation efforts but also to help lay the groundwork for systematic characterization and breeding efforts for these species of long-standing horticultural significance.

## Materials and Methods

### Germplasm collection, DNA isolation, sequencing, genotyping, and ploidy determination

A set of 138 cold-hardy *Actinidia* accessions (101 genotypes of tetraploid *A*. *arguta* [2n = 4x = 116], 2 genotypes of hexaploid *A*. *arguta* [2n = 6x = 174], 28 genotypes of *A*. *kolomikta* [2n = 2x = 58], and 7 genotypes of *A*. *polygama* [2n = 2x = 58]) were assembled from USDA National Clonal Germplasm Repositories, nurseries, and private growers. Complete passport information for each accession [e.g. genotype name, source, material received, reported and observed gender information, geographic provenance (if known), ploidy level, etc.] can be found in S1 Table.

For each accession, genomic DNA was isolated from ~1 g of fresh young leaves using a modified CTAB protocol, subsequently cleaned with a spin column (Zymo Research, Genomic DNA Clean & Concentrator^™^-10), and then multiplexed (6–10 bp barcodes) into genotyping-by-sequencing (GBS) libraries using a two enzyme (*PstI*-*MspI*) protocol [19]. The libraries were sequenced using 150 bp paired-end (PE) reads on an Illumina 2500 HiSeq platform at the Hubbard Center for Genome Studies (University of New Hampshire), and the raw FASTQ files were generated using CASAVA 1.8.3 [20]. All parsed, high-quality, PE reads are available in the NCBI Sequence Read Archive (SRA Accession numbers SRR3234098—SRR3234199 and SRR4308494 for *A*. *arguta*, SRR3234070—SRR3234097 for *A*. *kolomikta*, and SRR3234063—SRR3234063 for *A*. *polygama*). The compositions of the three GBS libraries used in this study, as well as all individual accession barcode assignments, can also be found in S1 Table.

The CASAVA-processed raw sequence data were submitted to version 2.0 of the GBS SNP-Calling Reference Optional Pipeline (GBS-SNP-CROP) [21] for sequence analysis, and genotyping. Demultiplexing and stringent quality filtering of the raw reads were carried out as explained in detail in the pipeline documentation (see https://github.com/halelab/GBS-SNP-CROP), and all recommended ploidy-specific parameters were used for intraspecific genotyping. For the initial combined, interspecific analysis, SNPs were called using the genotyping parameters that corresponded to the ploidy level of the majority of the 138 accessions evaluated (i.e. tetraploid). For complete details of the GBS-SNP-CROP command lines used in this study, including all specified pipeline parameters, please see S1 Text. For all downstream diversity analyses, we retained only those SNPs located within centroids (i.e. consensus GBS fragments) containing a single SNP, hereafter referred to as simplex SNPs.

Ploidy level across the diverse *Actinidia* genus varies widely, and recent studies have underscored the need to inspect the ploidy levels of individual accessions rather than assuming them on the basis of taxonomy [22]. *A*. *arguta* in particular is notably variable, with observed ploidy levels ranging from 4x to 10x [23, 24]. To assess the ploidy levels of the 138 accessions in this study, each species was handled separately. First, the centroids comprising each species-specific mock reference were mapped to the available *A*. *chinensis* var. *chinensis* reference genome [25]; and only simplex SNPs within centroids that aligned to unique positions in that reference were retained. The numbers of SNPs that passed these criteria were 1391, 1933, and 1318 for *A*. *arguta*, *A*. *kolomikta*, and *A*. *polygama*, respectively. For each accession, allele depth ratios were then calculated for all heterozygous SNPs; and the distribution of those ratios was plotted. As argued in other studies [26], such a distribution will exhibit a single peak (at 0.5) for diploid (2x) genomes, three local maxima (at 0.25, 0.5, and 0.75) for tetraploid (4x) genomes, five local maxima (at 0.17, 0.33, 0.5, 0.66, and 0.83) for hexaploid (6x) genomes, and so on. Such patterns were indeed observed in this case (see S2 Fig), permitting the inference of ploidy levels. In addition, ploidy estimations based on previously published flow cytometry analysis [27] were available for 38 of the accessions in this study and confirmed our findings. In five cases, the distribution of allele depth ratios were unclear; but ploidy could be inferred based on genotypic redundancy with other lines (see S1 Table).

### Characterizing genetic diversity

To characterize genetic diversity both within and across the collections of the three species in this study, we first used the GenAIEx 6.5.01 software [28] to generate descriptive parameters such as the number of effective alleles (N_E_), the Minor Allele Frequency (MAF), the observed (H_O_) and unbiased expected heterozygosities (H_E_) [29], and the intrapopulation fixation index (F_IS_). To estimate the pairwise genetic dissimilarities between accessions, we employed a modified Gower’s Dissimilarity Coefficient (GD) [30]. Ranging from 0 to 1, GD assesses accession dissimilarity by quantifying the identity-by-state (IBS) of all bi-allelic SNPs, according to:

$$D_{Gower}\left(x,y\right)=1-\left(\frac{\sum_{i=1}^{m}s_{i}w_{i}}{\sum_{i=1}^{m}w_{i}}\right)$$

where s_i_ = 1 if the genotypes are the same at SNP_i_, 0.5 if the genotypes differ by one allele at SNP_i_ (i.e. heterozygote vs. homozygote), and 0 if the genotypes differ by both alleles at SNP_i_ (i.e. primary homozygote vs. secondary homozygote); and w_i_ = 1 if both accessions are genotyped SNP_i_ and 0 if either accession lacks an assigned genotypic state (e.g. due to low coverage).

As formulated above, the GD is a faithful metric of pairwise allelic IBS between diploid accessions (e.g. *A*. *kolomikta*, *A*. *polygama*), provided the SNP-containing regions are single copy in the genome. By disallowing multiple alignments to the mock reference and permitting only simplex markers to pass for downstream diversity analysis, the genotyping pipeline rigorously selects for such high-confidence SNPs. For polyploid accessions (e.g. *A*. *arguta*), the above GD formulation is also accurate, provided the SNPs used are not only single copy but also effectively diploidized (i.e. they are polymorphic within, rather than between, homoelogous sub-genomes). Such markers are identifiable via inspection of their allele depth ratios, for example being consistently ~0.25 within heterozygous tetraploid accessions. Using such a subset of 382 diploidized SNPs for the *A*. *arguta* accessions, we detected the exact same population sub-structure and groups of redundant genotypes as when ignoring this ploidy complication and calculating GD based on all 2,964 simplex SNPs.

To further test if the simple, modified GD defined above functions reliably as a distance metric for 4x accessions, we compared its performance to that of a slightly modified version specifically tailored to that level of ploidy. Specifically, the heterozygous IBS class, previously coded as 0.5 (one copy of each allele), was split into three heterozygous IBS classes based on allele depth ratios (0.25, 0.5, and 0.75) representing the three possible heterozygous states of homoelogous loci (3:1, 1:1, and 1:3). Again, as with the diploidized set of SNPs used above, this 4x-specific coding of the heterozygote classes returned the same population sub-structure and groups of redundant genotypes as the simpler GD expression. For basic diversity characterization and the identification of redundant accessions, the GD formulation described above is a robust metric and is therefore used uniformly throughout this study.

### Determining the dissimilarity threshold for declaring redundant accessions

Because of its implications in terms of resource use efficiency of both curation and breeding efforts, one of the most important objectives of germplasm characterization is the identification of redundant materials/genotypes within collections. While redundant genotypes should in theory exhibit a pairwise GD = 0, the existence of both sequencing error and genotyping error due to imperfect sampling (i.e. GBS fragment representation bias) means that, in practice, perfect genotypic similarity among replicated accessions is rarely observed. In order to declare identity between two accessions with a certain confidence, it is therefore necessary to use the available sequence data to set a GD maximum threshold, below which two accessions are declared to be identical.

To determine the species-appropriate values of this threshold for this study, we pursued three different strategies. First, we carried out duplicate DNA isolations from three different genotypes, prepared them as separate samples within the same GBS library, and sequenced them in the same Illumina lane (i.e. biological replicates). Second, we isolated duplicate DNA samples from seven different genotypes, split the duplicates between two different library preparations, and sequenced the libraries on two different Illumina lanes (i.e. technical replicates). Finally, to assess the effect of imperfect sampling, we selected three genotypes from three different sequencing libraries (one genotype per library) and randomly sampled, with replacement, 50% of their paired reads, creating ten different sub-samples for each genotype. These sub-samples were mapped to species-specific Mock References (for details, please see Stage 2 of the GBS-SNP-CROP user manual at https://github.com/halelab/GBS-SNP-CROP/wiki) and genotyped using the GBS-SNP-CROP pipeline v.2.0 [21]. For all three cases described above, GD's were calculated for each pairwise genotype comparisons (i.e. between biological replicates, between technical replicates, and between sub-samples). Based on these results, 99% confidence intervals were constructed to identify appropriate dissimilarity thresholds for declaring accession redundancy within the germplasm collection. The first two strategies (biological and technical replication) were employed only for *A*. *arguta* and *A*. *kolomikta*, while the third was used for all three *Actinidia* species in the study.

### Selecting the hierarchical clustering method

To generate cladograms representing the relationships among accessions in the collection, we evaluated all eight different hierarchical clustering methods available through the hclust() function in R [31] in order to choose the algorithm most appropriate to our data (both inter- and intraspecific clustering). The hierarchical clustering methods evaluated were *Ward*.*D* and *Ward*.*D2* [31, 32]; *single*, *complete*, and *average* (i.e. UPGMA—Unweighted Pair Group Method with Arithmetic Mean) [33]; as well as variations of the UPGMA method, such as *mcquitty* (WPGMA) [34], *median* (WPGMC), and *centroid* (UPGMC). Selection of the most appropriate method was based on consideration of their relative Cophenetic Correlation Coefficients (CCC) [35], estimated using 10,000 bootstraps.

### Analyzing population genetic structure

We used the R package 'Pvclust' [36], with some modifications to incorporate GD estimation, to produce bootstrapped (n = 1,000) cladograms with p-values indicating the stability of nodes (i.e. monophyletic groups). In addition to performing inter- and intraspecific genetic distance-based hierarchical clusterings, we also assessed the overall genetic structure of the germplasm collection via multivariate Principal Component Analyses (PCA) through the dudi.pca() function in R (package 'adegenet') [37].

To determine the number of distinct clusters, or sub-populations (K), underlying the intraspecific population structures of the *A*. *arguta* and *A*. *kolomikta* collections, we applied both the Discriminant Analysis of Principal Components (DAPC) procedure [38] as well as the K-Means clustering procedure [39]. The former analysis was executed using the find.clusters() function in R (package 'adegenet') [37] and the latter using the software package GenoDive [40]. In both analyses, a range of clusters was evaluated (K = 2 to 10) and the optimal number chosen based on minimizing the associated Bayesian information criterion (BIC). Finally, we relied on the GenAlEx 6.5.01 software [28] to perform an analysis of molecular variance (AMOVA), using a total of 10,000 permutations, to assess the hierarchical partitioning of genetic variation among the detected groups. All analyses of population structure (i.e. DAPC, K-Means, and AMOVA) were performed after identifying and culling redundant genotypes from the dataset, retaining only a single representative accession for each redundant group.

## Results

### Genotyping

Based on a combination of previously published flow cytometry data [27] and the observed distributions of heterozygous allele depth ratios in this study, all *A*. *kolomikta* and *A*. *polygama* accessions in this collection were confirmed to be diploid. Similarly, nearly all of the 103 *A*. *arguta* accessions in this study were found to be tetraploid, with the exception of two redundant hexaploid genotypes [cvs. 'Issai' (PI 667909) and 'Issai small fruit variant' (PI 617116)]. While this near uniformity of ploidy level among the US collection of *A*. *arguta* germplasm stands in contrast to the widely varying levels observed in some natural populations in the species' center of diversity [22–24], it is an understandable result given the relatively limited importation of wild germplasm into the US to date. Based on these results, diploid parameters were used to call SNPs within both *A*. *kolomikta* and *A*. *polygama*, while tetraploid parameters were used to call SNPs within *A*. *arguta*. Additionally, because more than 70% of the 138 accessions in the study are 4x, tetraploid parameters were also used to call SNPs for the initial, combined interspecific analysis (see S1 Text for more details).

After culling all SNPs from Mock Reference centroids containing more than one polymorphism, the numbers of high-confidence simplex SNPs retained for downstream analyses were 2,964 (mean depth D = 44.0) for *A*. *arguta*, 3,425 (D = 33.2) for *A*. *kolomikta*, and 2,037 (D = 47.6) for *A*. *polygama*. These sets of SNPs were called using 476.8, 130.8, and 19.2 million PE high quality reads for *A*. *arguta*, *A*. *kolomikta*, and *A*. *polygama*, respectively. In addition to the passport data for all accessions in the study, S1 Table reports the total number of PE reads for each accession. All three species exhibited similar values of overall loci heterozygosity, homozygosity, and missing data, with the intraspecific averages being 33.8% (Hetero), 59.6% (Homo), and 6.5% (NA) (Table 1).

**Table 1:** Summary data characterizing the sets of SNPs used for all three species together (interspecific analysis) and for each species separately (intraspecific analyses).

Species	N^a^	PE reads^b^	SNPs^c^	D^d^	D > 20^e^	Hetero^f^	Homo^g^	NA^h^
*Interspecific analysis*								
*A*. *arguta*								
*A*. *kolomikta*	138	626,806,096	1,040	108	100	17.07	68.92	14.01
*A*. *polygama*								
*Intraspecific analyses*								
*A*. *arguta*	103	476,836,814	2,964	44.04	75.12	33.86	58.00	8.12
*A*. *kolomikta*	28	130,774,076	3,425	33.22	57.66	30.14	64.33	5.51
*A*. *polygama*	7	19,195,206	2,037	47.57	74.57	37.53	56.59	5.87
Average	—	208,935,365	2,808	41.61	69.11	33.84	59.64	6.50

^a^ Number of genotypes (i.e. accessions) sampled

^b^ Number of high quality, paired-end (PE) reads used to call SNPs

^c^ Number of SNPs called after imposing all genotyping criteria and subsequent filters

^d^ Average read depth (i.e. average number of independent supporting GBS fragments) for each called SNP

^e^ Percentage of called SNPs with an average read depth of at least 20

^f^ Percentage of heterozygous genotype calls

^g^ Percentage of homozygous genotype calls

^h^ Percentage of missing cells (i.e. no genotype assigned for a given SNP-accession combination)

### Identifying redundant accessions

The average GD between biological replicates prepared within the same library and sequenced within the same Illumina lane was found to be 0.0004 and 0.0009 for *A*. *arguta* and *A*. *kolomikta*, respectively (Table 2). For technical replicates (i.e. the same DNA samples prepared in two separate libraries and sequenced within two different Illumina lanes), the average pairwise GD increased roughly 7.2 times for tetraploid *A*. *arguta* (GD = 0.0029) and 6.4 times for diploid *A*. *kolomikta* (GD = 0.0058). These results indicate that, on average, genotyping error increases due to variation generated by different library preparations and sequencing runs. Consequently, in the absence of conflicting independent evidence (e.g. differential phenotypes), technical replicate thresholds should be considered more appropriate than biological replicate thresholds when flagging redundant genotypes in a resource-limited curation program.

**Table 2:** The mean Gower dissimilarity coefficients (GD's) and 99% confidence thresholds (GD_99%_, in parentheses) generated via three different strategies to assess and declare genotypic redundancy.

Species	Strategy		
	Biological Replicates	Technical Replicates	Read Sampling
*A*. *arguta*	0.0004 (0.0024)	0.0029 (0.0046)	0.0007 (0.0017)
*A*. *kolomikta*	0.0009 (0.0036)	0.0060 (0.0112)	0.0017 (0.0029)
*A*. *polygama*	--	--	0.0029 (0.0045)

Shapiro-Wilk tests for the intraspecies sets of technical replicate GD values revealed normal distributions of those values for both *A*. *arguta* (W = 0.970; p-value = 0.900) and *A*. *kolomikta* (W = 0.930; p-value = 0.556); therefore, normal probability densities were used to determine 99% confidence thresholds for declaring genotypic redundancy within each species. Specifically, since the extensive dataset used in this study is comprised of sequence data from multiple library preparations and Illumina lanes, these species-specific thresholds were based on confidence intervals from the mean GDs between *technical* replicates, resulting in 99% thresholds for redundant accessions (GD_99%_) of 0.0046 and 0.0112 for *A*. *arguta* and *A*. *kolomikta*, respectively.

In comparison, the read sampling strategy resulted in 99% GD thresholds of 0.0017 for *A*. *arguta* and 0.0029 for *A*. *kolomikta*, thresholds that are on average 0.75 and 0.30 times less than the estimated GD_99%_ thresholds based on biological and technical replicates, respectively (Table 2). Based on these relationships, and using the results of the read sampling strategy applied to *A*. *polygama* accessions, we estimated a GD_99%_ threshold of 0.0150 (i.e. 0.0045 / 0.30) for declaring redundancy among *A*. *polygama* accessions. In S2 Table, we report diagonal matrices showing the pairwise GD's across all 138 accessions in the study (lower diagonal) and the number of SNPs used to estimate those pairwise dissimilarities (upper diagonal), all based on the interspecific analysis using 1,040 SNPs.

### Clustering method selection and analyses of interspecific genetic structure

Evaluation of the eight different hierarchical clustering methods available through the R function hclust() was done for each species separately (intraspecific analyses) as well as for all accessions considered together (interspecific analysis). The UPGMA method (average) consistently produced higher CCC values for both analyses (S3 Fig) and was therefore chosen as the most appropriate overall hierarchical clustering method for cladogram construction. While the eight methods produced very similar values of CCC in the interspecific analyses, the variation among them was greater for the intraspecific analysis, especially for *A*. *arguta*, which helped clarify the selection of UPGMA as the most appropriate method.

**Fig 1:**
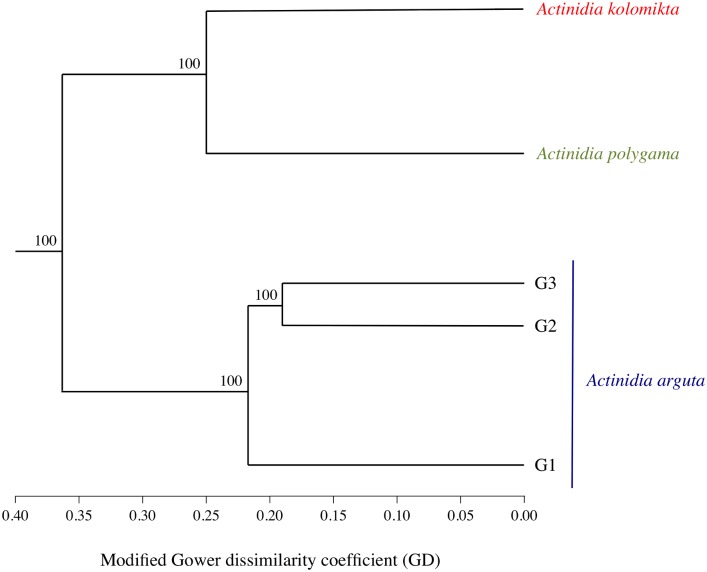
Interspecific UPGMA cladogram showing the genetic relationships, based upon a modified Gower’s dissimilarity coefficient (GD), among the three *Actinidia* species evaluated.

The resultant interspecific hierarchical clustering analysis (UPGMA) revealed clear differentiation among the three species, with *A*. *kolomikta* and *A*. *polygama* found to be more closely related to each other than to *A*. *arguta*. Based on the 1,040 high-confidence SNPs used for this interspecific analysis, *A*. *kolomikta* accessions are, on average, 25% dissimilar from those of *A*. *polygama*; and both species are more than 35% dissimilar from *A*. *arguta* genotypes (Fig 1). The interspecific PCA analysis corroborates these clear species-specific groups, with the first and second axes accounting for 40.94% and 6.95% of the total variation, respectively (S4 Fig).

### Analysis of intraspecific genetic diversity and population structure

**Fig 2:**
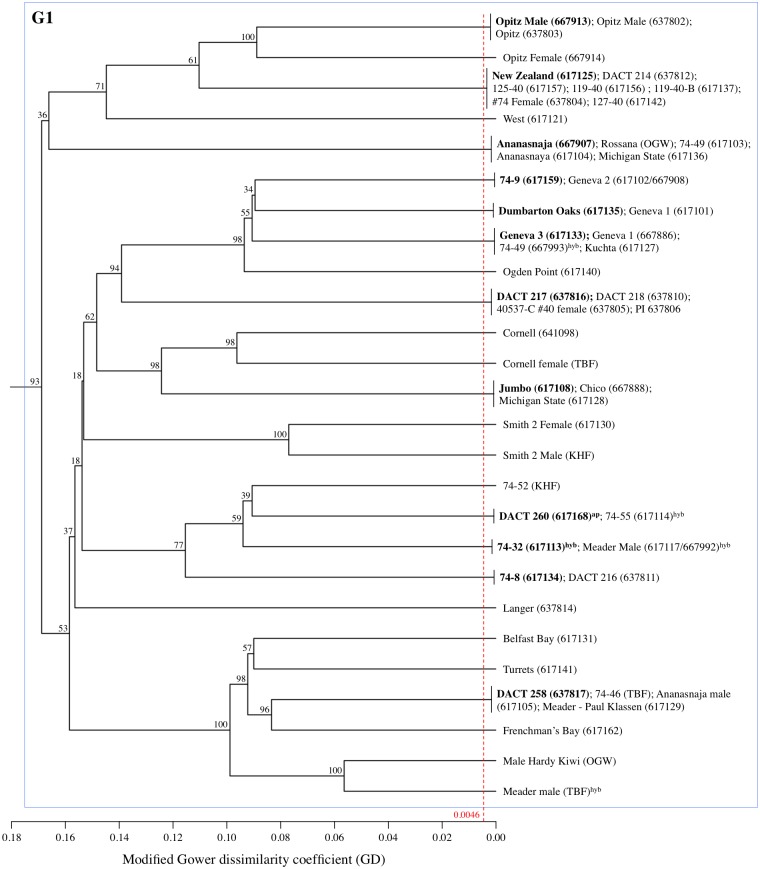
UPGMA cladogram for *A*. *arguta* sub-group G1. The red dashed line indicates the 99% confidence Gower dissimilarity threshold (GD = 0.0046) used to declare redundant accessions within this species. Within this sub-group alone, 12 redundant groups of genotypes were identified. Cladogram labels consist of an accession name followed by either its six-digit USDA plant introduction (PI) number, if part of the NPGS, or the initials of its non-USDA source (see S1 Table). Accessions in bold are the most read abundant genotypes within their respective redundant groups and are used to represent their groups on the full *A*. *arguta* intraspecific cladogram (S8 Fig). All accessions are tetraploid *A*. *arguta*, unless otherwise noted: ^hyb^ = putative unspecified interspecific hybrid with *A*. *arguta*; ^ap^ = putative *A*. *arguta* var. *purpurea*.

**Fig 3:**
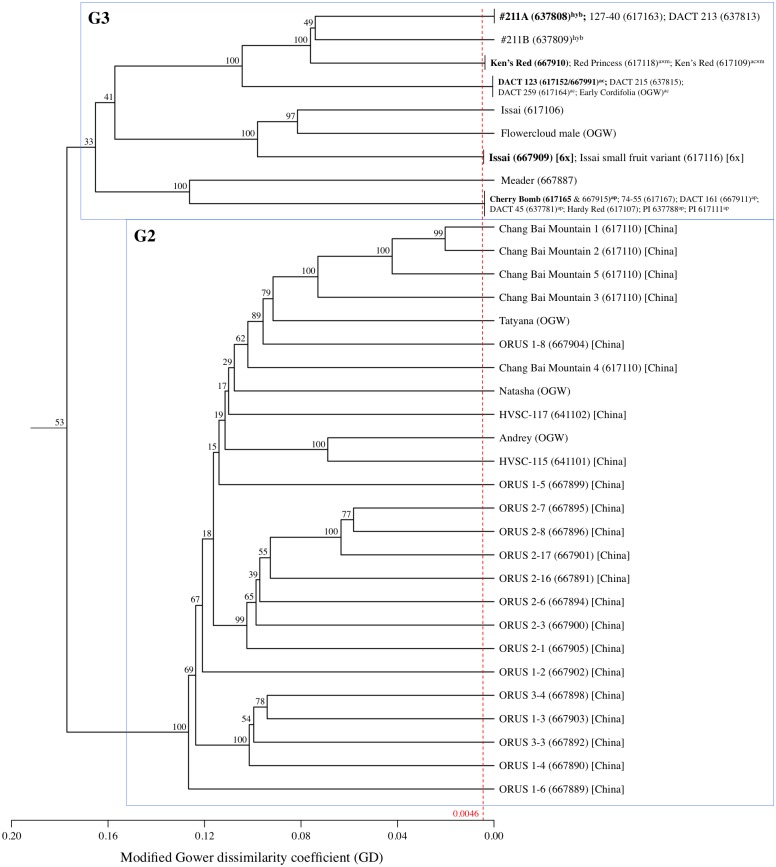
UPGMA cladogram for *A*. *arguta* sub-groups G2 and G3. The red dashed line indicates the 99% confidence Gower dissimilarity threshold (GD = 0.0046) used to declare redundant accessions within this species. No accession redundancy was found in G2, but 5 redundant groups of genotypes were identified within sub-group G3. Cladogram labels consist of an accession name followed by either its six-digit USDA plant introduction (PI) number, if part of the NPGS, or the initials of its non-USDA source (see S1 Table). Accessions in bold are the most read abundant genotypes within their respective redundant groups and are used to represent their groups on the full *A*. *arguta* intraspecific cladogram (S8 Fig). All accessions are tetraploid *A*. *arguta*, unless otherwise noted: [Ch] = Chinese provenance; [6x] = hexaploid; ^hyb^ = putative unspecified interspecific hybrid with *A*. *arguta*; ^ac^ = putative *A*. *arguta* var. *cordifolia*; ^ap^ = putative *A*. *arguta* var. *purpurea*; ^a×m^ = putative *A*. *arguta* × *A*. *melanandra* hybrid.

In terms of intraspecific genetic diversity, the unbiased expected heterozygosity (H_E_) was estimated to be 0.293 for *A*. *arguta*, 0.307 for *A*. *kolomikta*, and 0.400 for *A*. *polygama*. Low levels of inbreeding were observed (average F_IS_ = -0.089), an expected result given the dioecy of these three species. Minor allele frequencies (MAFs) were larger than 0.1 for, on average, 92.07% of all SNPs called; and the average GD between accessions within each species was 0.169, 0.210, and 0.295 for *A*. *arguta*, *A*. *kolomikta*, and *A*. *polygama*, respectively (Table 3; see also the *A*. *polygama* specific cladogram in S5 Fig). Both the DAPC and the K-Means analyses failed to identify clear sub-populations within the *A*. *kolomikta* and *A*. *polygama* groups (S6 Fig). For *A*. *arguta*, however, these analyses suggest the population is composed of three different sub-groups (Figs 2, 3 and S6 Fig).

**Table 3:** Population parameters characterizing the genetic diversity among and within the three collections of *Actinidia* species in this study.

Species	MAF^a^	N_E_^b^	H_O_^c^	H_E_^d^	GD^e^	F_IS_^f^
*Interspecific analysis*						
*A*. *arguta*						
*A*. *kolomikta*	90.14	1.001	0.109	0.079	0.185	-0.283
*A*. *polygama*						
*Intraspecific analysis*						
*A*. *arguta*	88.87	1.460	0.369	0.293	0.169	-0.193
*A*. *kolomikta*	87.34	1.487	0.320	0.307	0.210	-0.042
*A*. *polygama*	100.00	1.680	0.400	0.424	0.295	-0.031
Average	92.07	1.542	0.363	0.341	0.224	-0.089

^a^ Percentage of loci with a minor allele frequency greater than 10%

^b^ Average number of effective alleles per locus

^c^ Observed heterozygosity

^d^ Unbiased expected heterozygosity

^e^ Mean modified Gower dissimilarity coefficient

^f^ Inbreeding coefficient

Applying the GD dissimilarity threshold described above (i.e. GD_99%_ = 0.0046), a total of 17 different groups of redundant *A*. *arguta* accessions were identified within the study collection, each group containing between 2 and 8 distinct accessions (Figs 2 and 3). Of the total 103 *A*. *arguta* accessions evaluated, therefore, there are in fact only 60 unique genotypes warranting curation and consideration by breeding programs. DAPC further suggests that these 60 non-redundant genotypes can be assigned three main genetic pools, with the two main axes capturing approximately 25% of the total genotypic variation (S7 Fig). Originally composed of 54 putatively distinct accessions, *A*. *arguta* sub-group G1 exhibits the highest levels of redundancy (40 accessions implicated in 12 groups; Fig 2); and among its 26 non-redundant genotypes, sub-group G1 shows the lowest level of genetic diversity (H_E_ = 0.277). *A*. *arguta* sub-group G2, comprised of 25 accessions, contains no redundancy (Fig 3) and is characterized by a slightly higher genetic diversity (H_E_ = 0.305). Finally, sub-group G3, originally composed of 23 putatively distinct accessions, is shown to consist of 5 redundant groups and only 9 non-redundant genotypes (Fig 3). The genetic dissimilarity among the G3 non-redundant genotypes is relatively high (H_E_ = 0.302), a fact seen in both the cladogram and the *A*. *arguta* PCA plot (S7 Fig).

Based on an AMOVA of the three *A*. *arguta* sub-groups (Table 4), approximately 87% of the total detected variation in allele frequency is found within genotypes. Moving up the population structure hierarchy, 12% of the detected variation can be attributed to diversity among genotypes within sub-groups and only 1% to diversity among the three sub-groups. Even though the lowest BIC value indicates K = 3 as the most likely number of *A*. *arguta* sub-populations (S6 Fig), the low bootstrap value of 53% at the G2-G3 node (Fig 3; S8 Fig) fails to provide strong support for the their being distinct sub-groups. Indeed, when G2 and G3 are instead considered as a single group, overall F_ST_ increases from 0.014 (p-value = 0.041) to 0.020 (p-value = 0.009).

**Table 4:** Results of the AMOVA-based partitioning of the variance in allele frequencies within the collection of 60 non-redundant *A*. *arguta* genotypes.

Source of variation	df	SS	MS	p-value	Var	Var %
Among Groups	2	1,890.4	945.2	0.041	8.2	1.4
Among Genotypes	57	36,564.2	641.5	0.001	69.8	12.0
Within Genotypes	60	68,568.1	501.9	0.001	501.9	86.5

**Fig 4:**
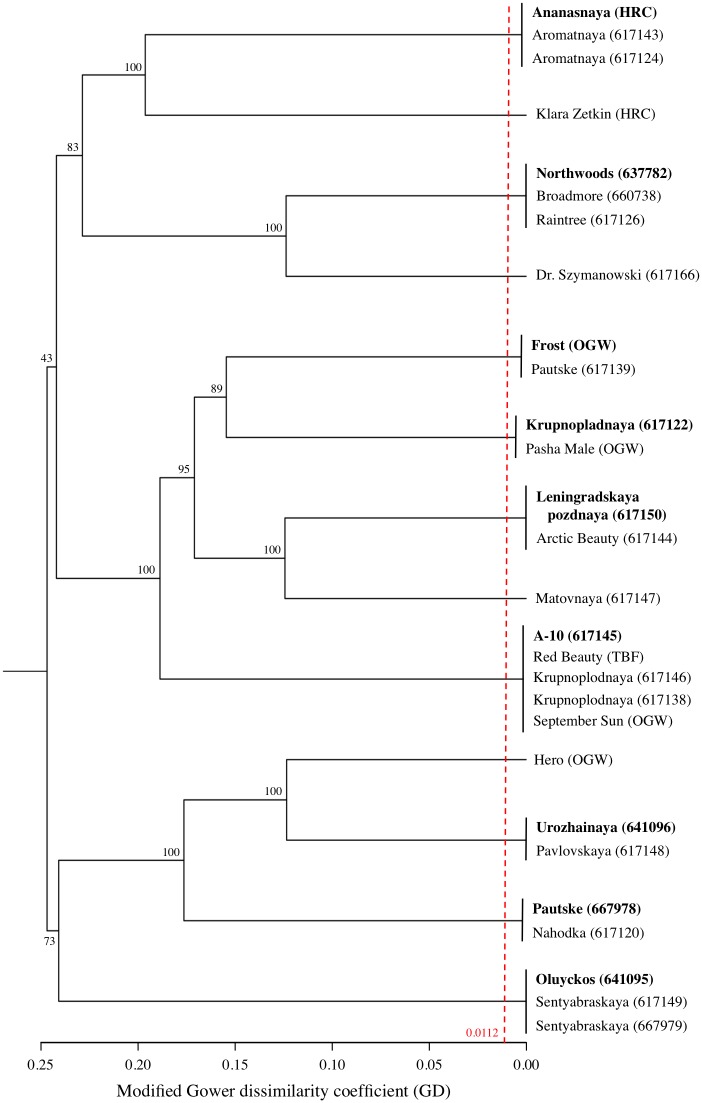
The *A*. *kolomikta* intraspecific UPGMA cladogram. The red dashed line indicates the 99% confidence Gower dissimilarity threshold (GD = 0.0112) used to declare redundant accessions within this collection. Nine groups of redundant accessions were found. Cladogram labels consist of an accession name followed by either its six-digit USDA plant introduction (PI) number, if part of the NPGS, or the initials of its non-USDA source (see S1 Table). Accessions in bold are the most read abundant genotypes within their respective redundant groups and are used to represent their groups on the bi-plot PCA analysis (S9 Fig).

A similar intraspecific analysis of *A*. *kolomikta* accessions revealed the existence of 9 different groups of redundant genotypes. Therefore, of the 28 accessions evaluated, only 13 distinct (non-redundant) genotypes should be considered for curation and oriented crosses in breeding programs (Fig 4). While the BIC values from the DAPC analysis were not sufficiently stable to suggest an optimal number of sub-groups (K value), the flagged (red arrow) bootstrap values ranging from 73% to 100% in Fig 4 suggest the possibility of three sub-groups. As shown in the PC1-PC2 biplot (S9 Fig), these potential subgroups of the 13 non-redundant *A*. *kolomikta* accessions are well discriminated by both PC axes (31.81% of the total variation).

## Discussion

As systematic assemblages of potentially useful genetic and phenotypic diversity, *ex situ* crop germplasm collections can serve as invaluable resources for plant breeders and other scientists working in the area of crop improvement, provided the characterization of such collections is sufficiently accurate to permit their effective use [41]. Depending on the type of collection required (e.g. seed vaults for small grains vs. living repositories for horticultural tree species), the cost of maintaining (or curating) plant genetic resource collections can vary greatly; but there is a need to increase resource-use efficiency in all cases. The strategy of selecting representative "core collections" is now widely followed as a means of increasing the efficiency of both curation and utilization efforts while preserving as much genetic diversity as possible of the entire collection [42]. For the US collections of the commercially promising species *A*. *arguta* and *A*. *kolomikta*, the baseline genotypic characterization necessary to achieve these goals is long overdue.

As shown by this investigation, the 140 convoluted years of cold-hardy *Actinidia* germplasm in the US have resulted in significant levels of redundancy within the USDA's National Plant Germplasm System (NPGS). For both species, there are numerous instances of the same genotype being maintained under different identifiers [e.g. *A*. *arguta* accessions 'Jumbo' (PI 617108) and 'Chico' (PI 667888); *A*. *kolomikta* accessions 'Raintree' (PI 617126), 'Broadmore' (PI 660738), and 'Northwoods' (PI 637782); etc.]. Accounting for such redundancies, there appears to be only 60 and 13 unique genotypes within the collections of 103 and 28 putatively different *A*. *arguta* and *A*. *kolomikta* genotypes, respectively, included in this study. For the more commercially promising species *A*. *arguta*, this amounts to a 68% over-estimation of accession diversity in the collection, requiring an additional 68% more resources to curate with no gain in collection diversity. For *A*. *kolomikta*, this over-estimation is an astounding 115%. Identifying such redundancies is critical not only in terms of curation efficiency but also, perhaps more importantly, for the resource-limited evaluation and use of these materials by breeding programs.

The variable extent of redundancy among accessions within the three *A*. *arguta* sub-groups (108% in Group 1, 144% in Group 2, and 0% in Group 3) is noteworthy and lends support to the idea that the convolution we observe today is the result of this species' long history of fragmented curation efforts among disparate institutional, private, and commercial actors. Group 3, for example, composed almost entirely of accessions collected by the USDA since 1999 and consistently maintained within the USDA system since their collection, exhibits no redundancy (Fig 3). This stands in stark contrast to Groups 1 and 2, both of which contain pre-1980's selections that entered the USDA collection, apparently multiple times under multiple names, by way of third parties. Many of the accessions in Group 1 (e.g. 'Ananasnaya,' 'Dumbarton Oaks,' 'Michigan State,' the 'Geneva' series, etc.) are, in fact, "re-discovered" vines with likely origins in the early (pre-1950's) ornamental *A*. *arguta* trade. Others were later selections (1950's-1970's) for fruit production (e.g. the '74' series from the USDA's Chico station, relinquished to the nursery trade; UNH's 'Meader' lines; etc.) that entered the current USDA via indirect means. Similarly, Group 2 consists of many selections that enjoyed at least some circulation within the post-1980's small fruit nursery trade (e.g. 'Issai,' 'Ken's Red', the '#211' lines, the cordifolia lines, etc.) before submission to the NPGS via third parties.

The overall population structure observed within the *A*. *arguta* collection may be explained, at least in part, by geographical provenance. It is clear from NPGS passport data that the USDA-collected and selected accessions in Group 3 (e.g. the 'Chang Bai Mountain,' 'ORUS,' and 'HVSC' lines) are all of Chinese origin. Generally speaking, such lines are characterized by pale-green to faintly pink petioles, earlier autumn leaf senescence, and increased cold tolerance as indicated by higher rates of survival and flowering following a winter with a -34°C minimum temperature (R. Guthrie, pers. observation). This stands in contrast to the more historic Group 1 accessions, likely derived from *A*. *arguta*'s earlier introductions to the US from Japan (e.g. '#74 Female' and 'Ogden Point'). In general, accessions in this group exhibit reddish to bright-red petioles, appear relatively less cold-tolerant than Group 3 lines, and contain vines producing some of the best flavored berries (e.g. 'Geneva 3', 'Dumbarton Oaks,' and 'Ogden Point'; data not shown). Group 2 is less easily explained in terms of geographic provenance, but its composition is interesting. Consisting entirely of pre-1990's selections made for fruit production (including pollinators), Group 2 brings together in a single clade the red-fleshed varieties (i.e. *A*. *arguta* var. *cordifolia* and *A*. *arguta* var. *purpurea*), red-fleshed putative hybrids with *A*. *melanandra* (e.g. 'Ken's Red'), other unspecified *A*. *arguta* hybrids (e.g. #211A), and ploidy variants (e.g. Issai). The discovery of distinct accessions with varying levels of ploidy sharing the name 'Issai' is not surprising, given previous reports of both 6x and 7x clones in other collections [43].

Unlike with *A*. *arguta*, no robust sub-groups were identified within the collection of *A*. *kolomikta* accessions. Genotypic analysis did reveal, however, a significant level of redundancy even among this relatively smaller collection, thereby indicating an opportunity to increase curation and utilization efficiency. In several cases, redundancy appears to be the result of the modern rebranding of known genotypes [e.g. 'Pautske' (PI 617139) and 'Krupnopladnaya' (PI 617122)] with US commerical nursery trade names [e.g. 'Frost' and 'Pasha Male' (One Green World)].

In addition to the cases of multiple identifiers being assigned to the same genotype, leading to significant levels of redundancy in these collections, there are also cases of different genotypes possessing the same identifier, clear cases of germplasm mix-ups over long history of these species in the US. Within the *A*. *kolomikta* collection (Fig 4), for example, the variety name 'Krupnoplodnaya' is shared by three different accessions. While two of these are female and genetically redundant (but with alternative spellings: PI 617146 and PI 617138), the third is not only a different genotype but also a different gender (PI 617122). In another case, the name 'Pautske' is shared between two genetically distinct accessions (PI 667978 and PI 617139). Similar examples abound within the *A*. *arguta* collection, with accession names 'Geneva 1,' 'Michigan State,' '127–40', 'Issai', "Meader Male', and others shared by distinct genotypes (Figs 2 and 3). Finally, although it was included primarily as an outgroup for this study, the small collection of *A*. *polygama* accessions was also found to exhibit both redundancy and clear error. As shown in S4 Fig, accessions 'UW-1' and 'DACT 310' are likely redundant; and accession 'NA 64534,' classified in the NPGS database as *A*. *arguta*, is revealed by its sequenced-based genotype to be an accession of *A*. *polygama*. The misclassification of *A*. *arguta* individuals as *A*. *polygama* is nothing new; in fact, confusion between these two species was quite common in the decades that followed *Actinidia*’s 1877 introduction to the US [44]. In the case of accession 'NA 64534,' the shoots possess a solid white pith characteristic of *A*. *polygama*, as opposed to the diagnostic brown chambered pith of *A*. *arguta*; therefore, field phenotyping confirms what the sequence data detected.

In terms of methodology, this study indicates that the bioinformatics pipeline GBS-SNP-CROP [21] can efficiently and cost-effectively identify redundant accessions, resolve closely-related species, and detect population sub-structure in the absence of a reference genome. Investigation into the patterns of variation in GD among intralibrary (biological) and interlibrary (technical) GBS replicates revealed that library preparation and lane-to-lane sequencing effects inflate the pairwise GD between identical genotypes. These effects should therefore be accounted for when deciding whether or not two lines are genetically distinct; moreover, the thresholds used for such decision-making can vary depending on the population and thus should be inferred empirically. As indicated here for *A*. *arguta* and *A*. *kolomikta*, it is possible that such thresholds may be estimable without the need for continuous investment in biological and/or technical replicates, provided: 1) An ability to approximate intralibrary GD error via read sub-sampling within an individual, as in this study; and 2) A relatively stable ratio of interlibrary-to-intralibrary GD error like the ones found in this study (~7.2 for *A*. *arguta*; ~6.4 for *A*. *kolomikta*). Whether or not these conditions hold true generally for other species, other sequencing platforms, and other variant-calling pipelines, remains a matter of investigation. For both breeding and germplasm curation programs, interlibrary effects are worth serious consideration, however, especially when single libraries and sequencing runs are impractical given the size of a collection and/or the need to compare newly acquired accessions with those analyzed previously.

## Conclusions

The accomplished USDA plant explorer David Fairchild had a longstanding interest in *Actinidia* spp. [45–47] and advocated, like others before him, for their horticultural potential [48]; yet it would be nearly 100 years after the initial introduction of *A*. *arguta* to the US before named kiwiberry varieties, generally selections no more that 2–3 generations from wild collected plants, entered the nursery trade. Half a century later, the horticultural potential of these species remains almost wholly untapped, while the germplasm ostensibly available for improvement in the US has eroded and become convoluted in our repositories and the nursery trade. Given the increasing threat of eradication of historic vines, particularly in the northeast, and the prohibition on the importation of new *Actinidia* accessions into the US due to concerns over Psa, the need to take stock of the surviving US collections of *A*. *arguta* and *A*. *kolomikta* is imperative. Through comprehensive molecular characterization, this study has revealed significant levels of redundancy in these collections while shedding light on the distribution and extent of diversity within these plant genetic resources. With this knowledge, not only can the resource efficiency of both breeding and curation programs be greatly improved, but systematic breeding strategies can begin to be developed.

## Supporting Information

S1 Table The list of the 103 *A*. *arguta*, 28 *A*. *kolomikta*, and 7 *A*. *polygama* accessions in this study. For each accession, the following information is provided: 1) Accession name; 2) Alternative names, spellings, or identifiers; 3) Source of material [USDA-ARS National Clonal Germplasm Repository in Corvallis, OR (Corvallis); USDA-ARS National Clonal Germplasm Repository in Davis, CA (Davis); University of Minnesota Landscape Arboretum Horticultural Research Center in Chanhassen, MN (HRC); KiwiHill Farm in Sidney, ME (KHF); Tripple Brook Farm in Southampton, MA (TBF); or One Green World in Portland, OR (OGW)]; 4) USDA Plant Introduction (PI) number(s), if assigned; 5) USDA Corvallis *Actinidia* (CACT) accession number, if assigned; 6) USDA Davis *Actinidia* (DACT) accession number(s), if assigned; 7) University of New Hampshire (UNH) ID, if assigned; 8) The kind of material received (dormant cutting, live cutting, or live plant); 9) Date of acquisition; 10) Reported gender; 11) Gender observed at UNH; 12) GBS library membership (1, 2, or 3); 13) GBS barcode assignment; 14) Number of high-quality paired-end (PE) reads used to call SNPs; 15) Number of SNPs called; and 16) Assigned NCBI Seqence Read Archive (SRA) number.
(XLSX)

S2 Table A summary matrix of all pairwise Gower dissimilarity coefficients (lower diagonal) and the numbers of SNPs used to estimate those coefficients (upper diagonal) for all 138 genotypes in this study. (XLSX)

S1 Text A complete log of the nine GBS-SNP-CROP command lines used in this study, with all parameters indicated. (PDF)

S1 Fig Timeline of the collection, curation, and dissemination of *Actinidia arguta* in the United States. A brief summary of events since the introduction of *A*. *arguta* into the United States in 1877, grouped according to institutional activities (left panels) and private/commercial activities (right panels).
(TIF)

S2 Fig Typical ploidy-specific distributions of allele depth ratios across heterozygous loci. Diploid (single peak at 0.5), tetraploid (three local maxima at 0.25, 0.5, and 0.75) and hexaploid (five local maxima at 0.17, 0.33, 0.5, 0.66, and 0.83) genomes are distinguishable based on their distinct patterns of peaks in these plots.
(TIF)

S3 Fig The Cophenetic Correlation Coefficient (CCC) values associated with each of the eight different hierarchical methods evaluated for each *Actinidia* species separately (intraspecific analyses) and all three together (interspecific analysis). The consistently strong performance of UPGMA (average) within the three species, and its clear superiority in the interspecific analysis, recommended it as the most suitable method for distance-based cladogram analysis in this study.
(TIF)

S4 Fig Principal component analysis of genotype data shows clear discrimination of the three cold-hardy *Actinidia* species in this study. (TIF)

S5 Fig The *A*. *polygama* intraspecific UPGMA cladogram. The red dashed line indicates the 99% confidence Gower dissimilarity threshold (GD = 0.0150) used to declare redundant accessions within this collection. One group of redundant accessions was found. Cladogram labels consist of an accession name followed by either its six-digit USDA plant introduction (PI) number, if part of the NPGS, or the initials of its non-USDA source (see S1 Table).
(TIF)

S6 Fig Bayesian information criterion (BIC) plotted as a function of the number of sub-groups within each *Actinidia* species evaluated. The optimum number of sub-groups that best explains the genetic structure within each species is considered to be the lowest value of K (i.e. minimum K) followed by an increase in BIC value. By these criteria, significant sub-structure is declared only for the *A*. *arguta* collection (K = 3).
(TIF)

S7 Fig Principal component analysis of genotype data indicates sub-structure among the non-redundant accessions of *Actinidia arguta*. The unbiased expected heterozygosities (H_E_) of the three *A*. *arguta* sub-groups are 0.277 (G1), 0.305 (G2), and 0.302 (G3). Subgroup G3 is notable for its dispersion among diverse accessions, including a ploidy variant (Issai– 6x), putative interspecific hybrids (Ken’s Red, #211A, #211B), a red-fleshed accession of *A*. *arguta* var. *purpurea* (Cherry Bomb), and a putative accession of *A*. *arguta* var. *cordifolia* (DACT 123).
(TIF)

S8 Fig The comprehensive *A*. *arguta* intraspecific UPGMA cladogram, showing the relationships among three sub-groups of non-redundant accessions. The red dashed line indicates the 99% confidence Gower dissimilarity threshold (GD = 0.0046) used to declare redundant accessions within this collection. The 17 bolded labels represent redundant groups of genotypes in which the bolded accession is the most read-abundant in the group. All accessions are tetraploid *A*. *arguta*, unless otherwise noted: [Ch] = Chinese provenance; [6x] = hexaploid; ^hyb^ = putative unspecified interspecific hybrid with *A*. *arguta*; ^ac^ = putative *A*. *arguta* var. *cordifolia*; ^ap^ = putative *A*. *arguta* var. *purpurea*; ^a×m^ = putative *A*. *arguta* × *A*. *melanandra* hybrid.
(TIF)

S9 Fig Principal component analysis of genotype data shows the relatedness among the thirteen non-redundant *A*. *kolomikta* germplasm accessions. (TIF)
