# Supplementary material for: GBS-Based Deconvolution of the Surviving North American Collection of Cold-Hardy Kiwifruit (Actinidia spp.) Germplasm
Source: PLoS One. 2017 Jan 26;12(1):e0170580. doi: 10.1371/journal.pone.0170580 (PMC5268759; doi:10.1371/journal.pone.0170580)
Supplement: S1 Text — (PDF) [file pone.0170580.s003.pdf]

S1 Text. A complete log of the nine GBS-SNP-CROP command lines used in this study, with all parameters indicated.

```
# GBS-SNP-CROP-1.pl
perl /path-to-workdir/GBS-SNP-CROP-1.pl -d PE -b barcodesIDs.txt -fq
L001 -s 1 -e 48 -enz1 TGCA -enz2 CGG


# GBS-SNP-CROP-2.pl
perl /path-to-workdir/GBS-SNP-CROP-2.pl -d PE -fq L001 -t 10 -ph 33 -
ad TruSeq3-PE.fa:2:30:10 -l 30 -sl 4:30 -tr 30 -m 32


# GBS-SNP-CROP-3.pl
perl /path-to-workdir/GBS-SNP-CROP-3.pl -d PE -b barcodesIDs.txt -fq
L001


# GBS-SNP-CROP-4.pl
perl /path-to-workdir/GBS-SNP-CROP-4.pl -d PE -b barcodeID.txt -rl
150 -pl 32 -p 0.01 -id 0.93 -t 10 -MR MockRefName


# GBS-SNP-CROP-5.pl
perl /path-to-workdir/GBS-SNP-CROP-5.pl -d PE -b barcodeID.txt -ref
MockRefName.MockRef.Genome.fasta -Q 30 -q 0 -f 2 -F 2308 -t 10 -Opt 0


# GBS-SNP-CROP-6.pl
perl /path-to-workdir/GBS-SNP-CROP-6.pl -b barcodeID.txt -out
SNPs.summary.txt


# GBS-SNP-CROP-7.pl – A. kolomikta and A. polygama (diploid species)
perl /path-to-workdir/GBS-SNP-CROP-7.pl -in SNPs.summary.txt -out
SNPs.call.txt -mnHoDepth0 5 -mnHoDepth1 20 -mnHetDepth 3 -altStrength
0.962 -mnAlleleRatio 0.25 -mnCall 0.75 -mnAvgDepth 4 -mxAvgDepth 200

# GBS-SNP-CROP-7.pl – A. arguta (tetraploid species)
perl /path-to-workdir/GBS-SNP-CROP-7.pl -in SNPs.summary.txt -out
SNPs.call.txt -mnHoDepth0 11 -mnHoDepth1 48 -mnHetDepth 3 -
altStrength 0.9 -mnAlleleRatio 0.1 -mnCall 0.75 -mnAvgDepth 4 -
mxAvgDepth 200


# GBS-SNP-CROP-8.pl
perl /path-to-workdir/GBS-SNP-CROP-8.pl -in SNPs.call.txt -out
SNP.Rmatrix -b barcodesIDs.txt -formats R
```
