# Supplementary figures and images for: GBS-Based Deconvolution of the Surviving North American Collection of Cold-Hardy Kiwifruit (Actinidia spp.) Germplasm
Source: PLoS One. 2017 Jan 26;12(1):e0170580. doi: 10.1371/journal.pone.0170580 (PMC5268759; doi:10.1371/journal.pone.0170580)

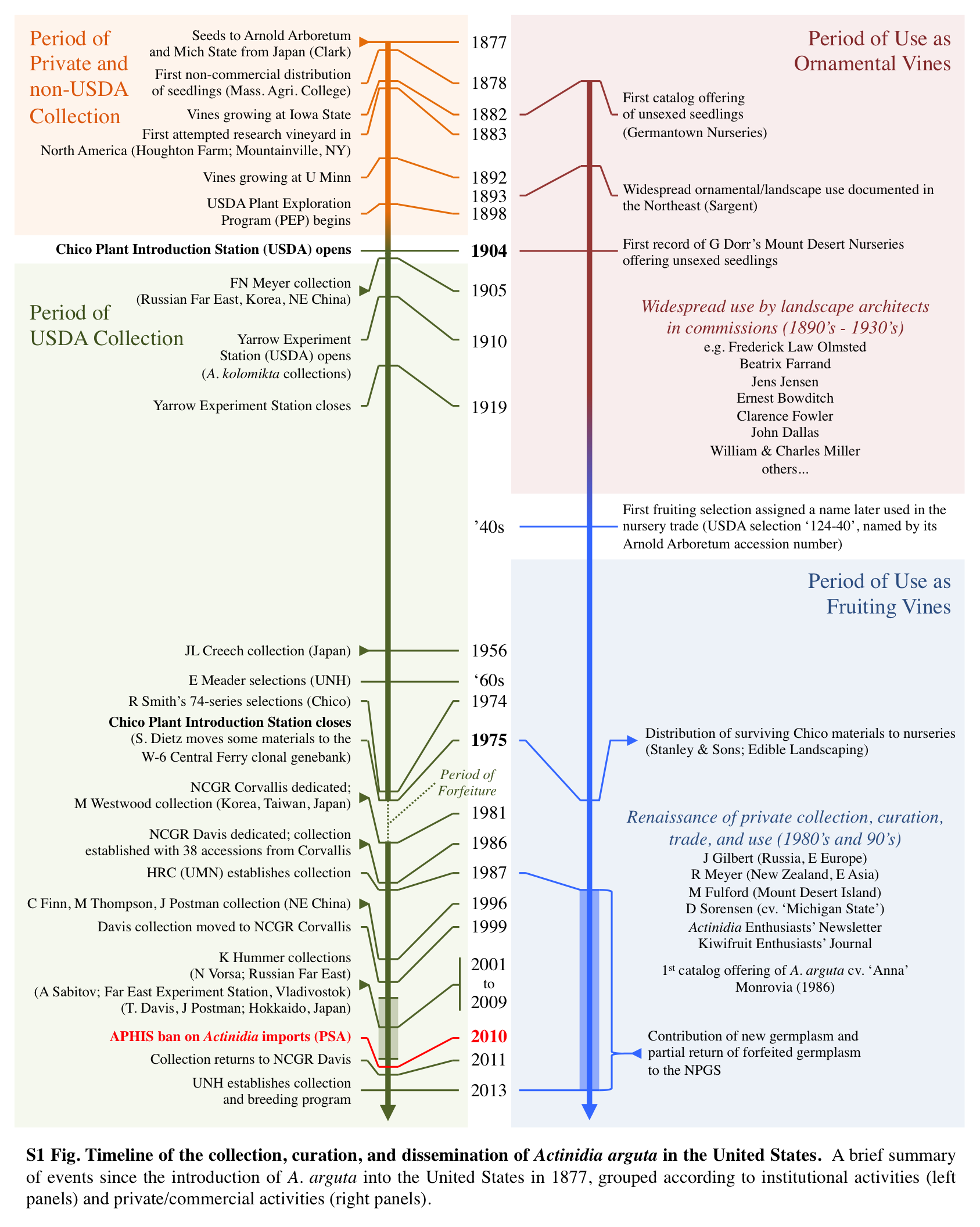

Supplement: S1 Fig — A brief summary of events since the introduction of A. arguta into the United States in 1877, grouped according to institutional activities (left panels) and private/commercial activities (right panels). (TIF) [file pone.0170580.s004.tif]

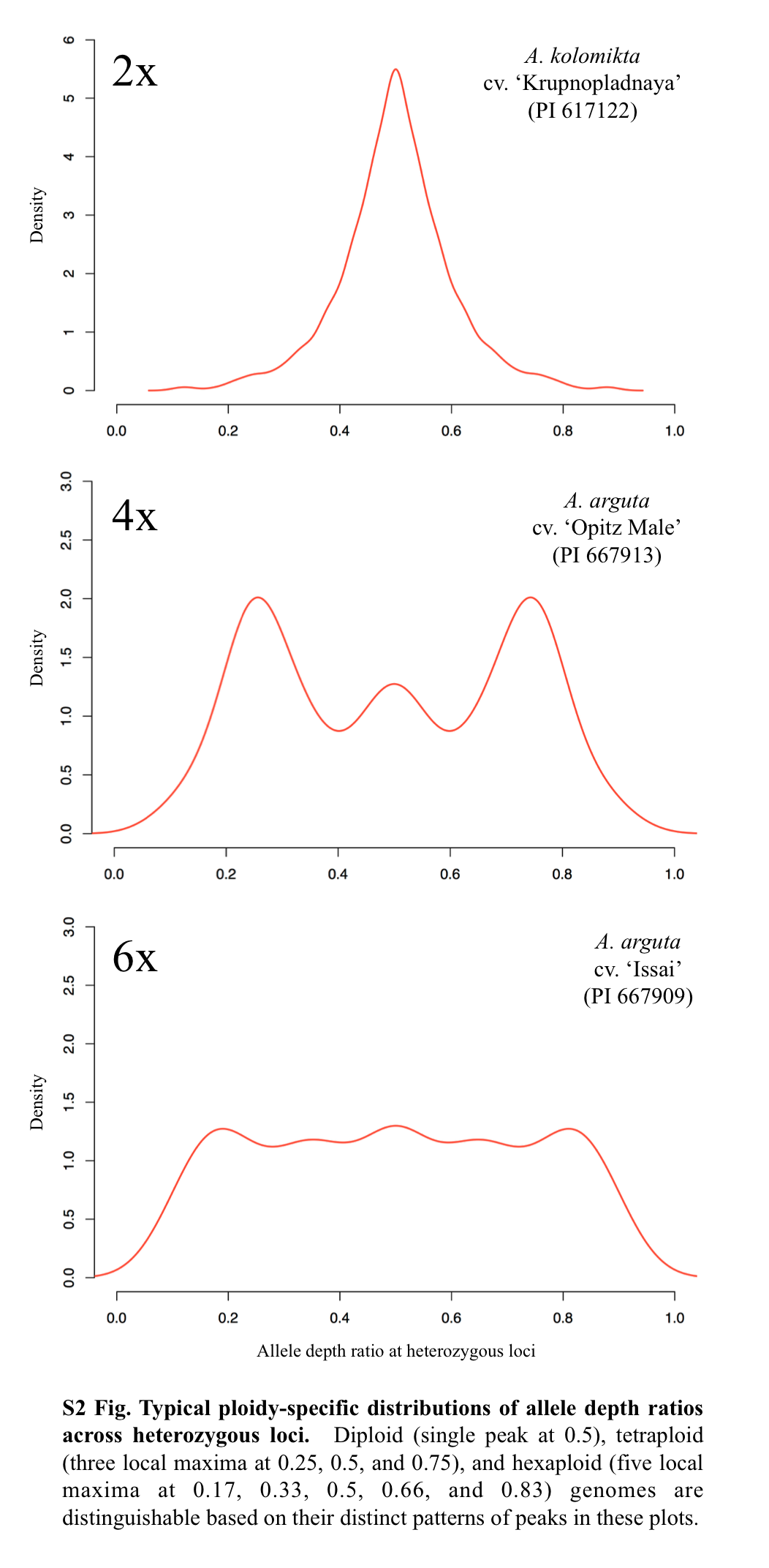

Supplement: S2 Fig — Diploid (single peak at 0.5), tetraploid (three local maxima at 0.25, 0.5, and 0.75) and hexaploid (five local maxima at 0.17, 0.33, 0.5, 0.66, and 0.83) genomes are distinguishable based on their distinct patterns of peaks in these plots. (TIF) [file pone.0170580.s005.tif]

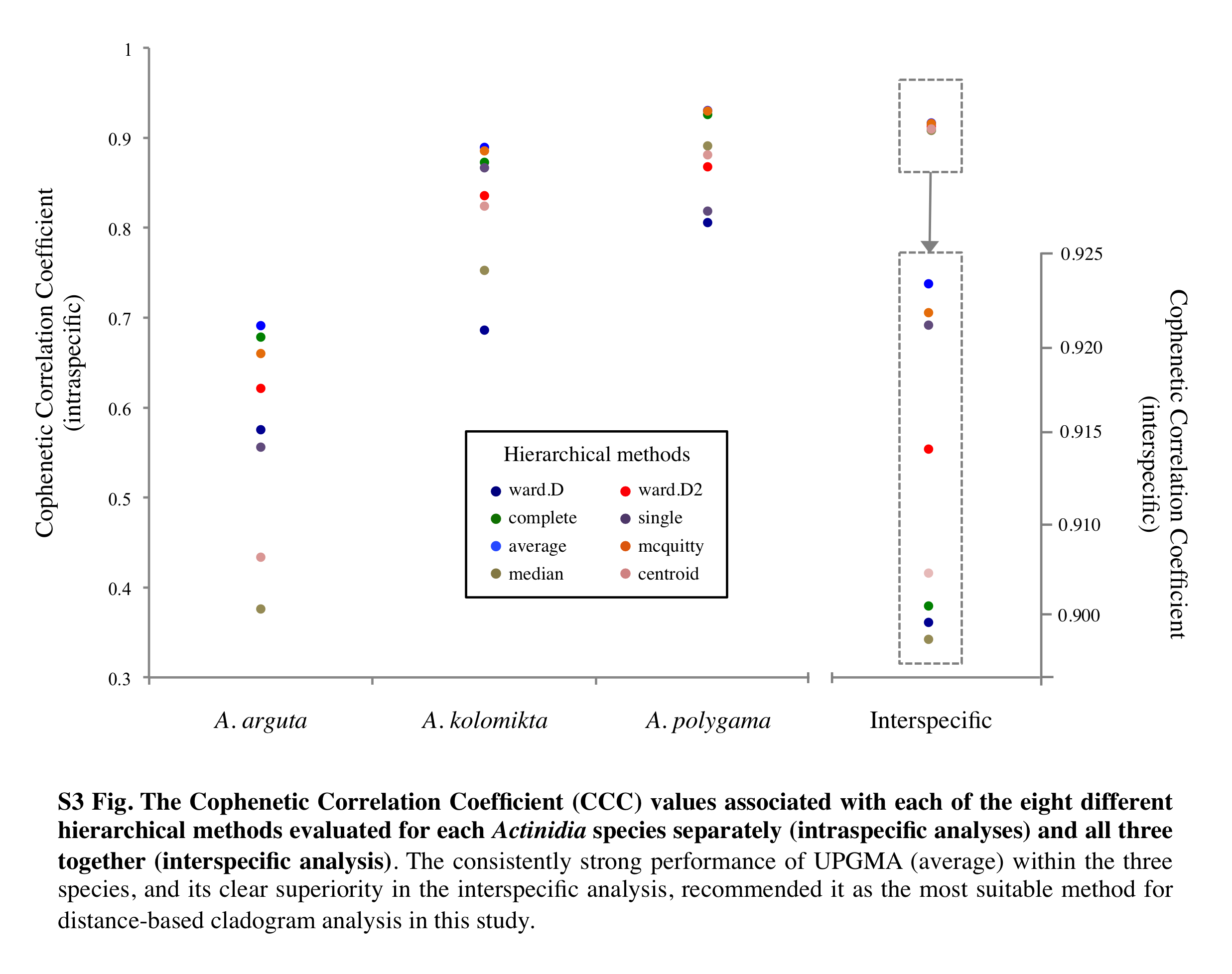

Supplement: S3 Fig — The consistently strong performance of UPGMA (average) within the three species, and its clear superiority in the interspecific analysis, recommended it as the most suitable method for distance-based cladogram analysis in this study. (TIF) [file pone.0170580.s006.tif]

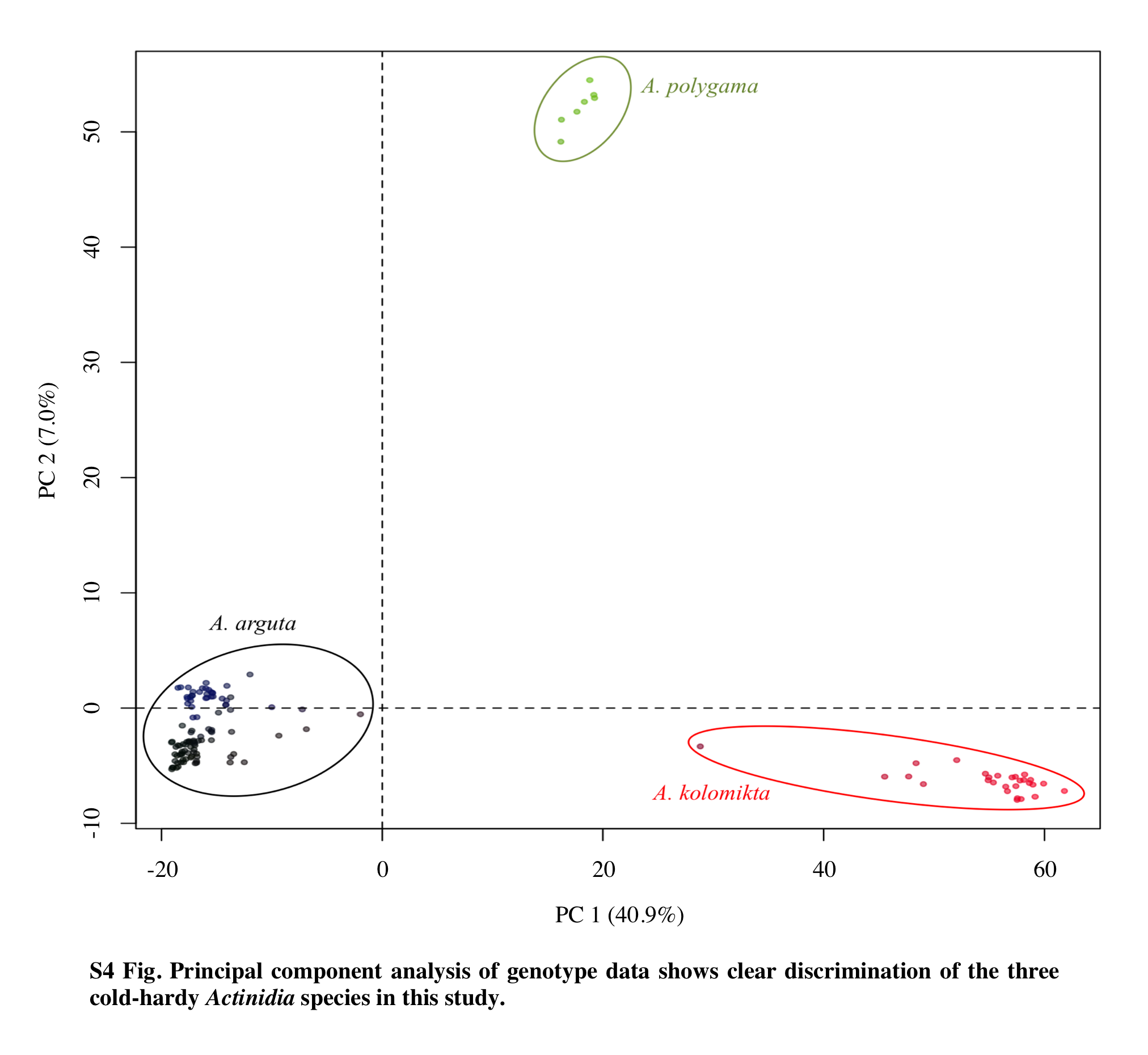

Supplement: S4 Fig — (TIF) [file pone.0170580.s007.tif]

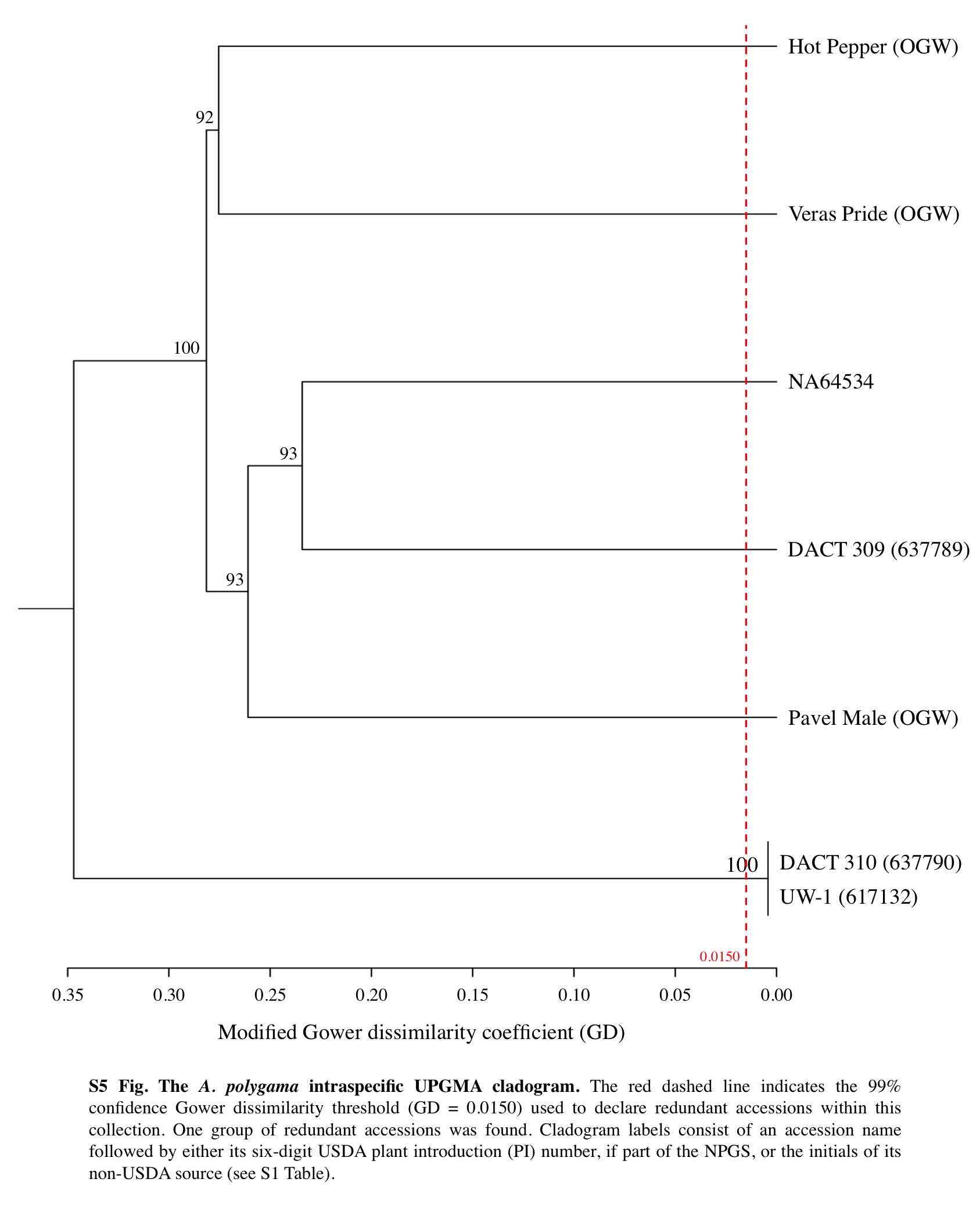

Supplement: S5 Fig — The red dashed line indicates the 99% confidence Gower dissimilarity threshold (GD = 0.0150) used to declare redundant accessions within this collection. One group of redundant accessions was found. Cladogram labels consist of an accession name followed by either its six-digit USDA plant introduction (PI) number, if part of the NPGS, or the initials of its non-USDA source (see S1 Table). (TIF) [file pone.0170580.s008.tif]

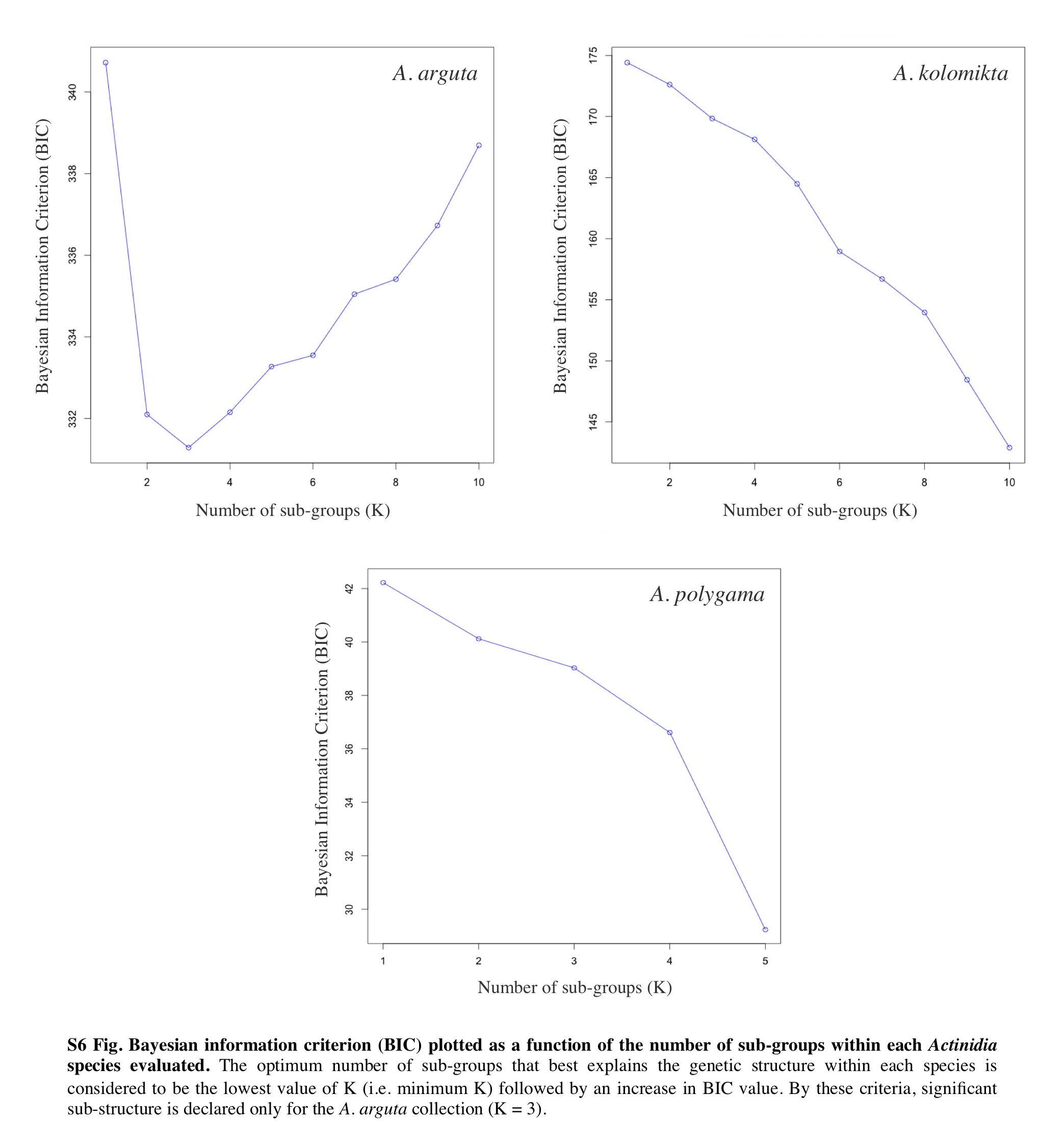

Supplement: S6 Fig — The optimum number of sub-groups that best explains the genetic structure within each species is considered to be the lowest value of K (i.e. minimum K) followed by an increase in BIC value. By these criteria, significant sub-structure is declared only for the A. arguta collection (K = 3). (TIF) [file pone.0170580.s009.tif]

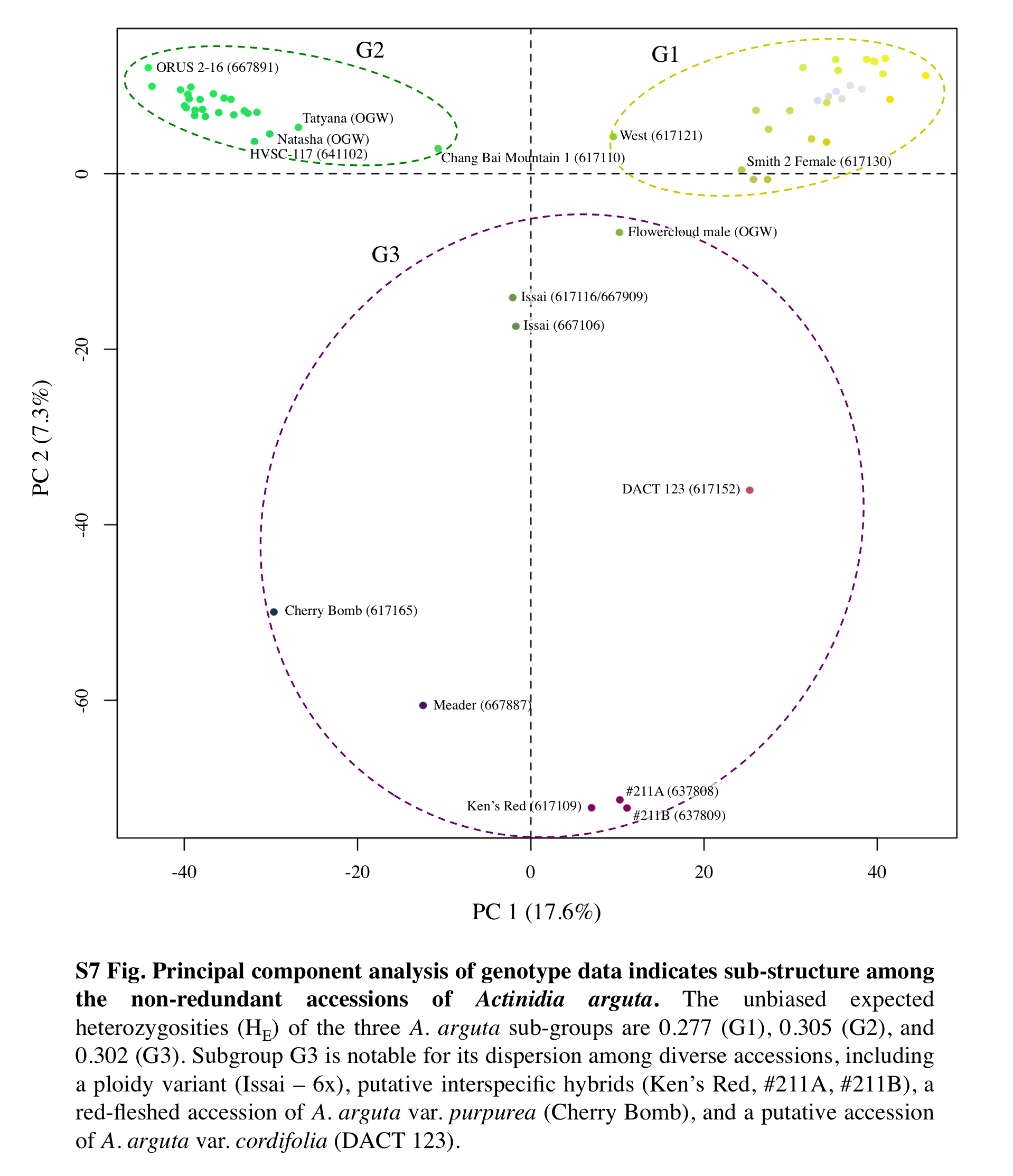

Supplement: S7 Fig — The unbiased expected heterozygosities (HE) of the three A. arguta sub-groups are 0.277 (G1), 0.305 (G2), and 0.302 (G3). Subgroup G3 is notable for its dispersion among diverse accessions, including a ploidy variant (Issai– 6x), putative interspecific hybrids (Ken’s Red, #211A, #211B), a red-fleshed accession of A. arguta var. purpurea (Cherry Bomb), and a putative accession of A. arguta var. cordifolia (DACT 123). (TIF) [file pone.0170580.s010.tif]

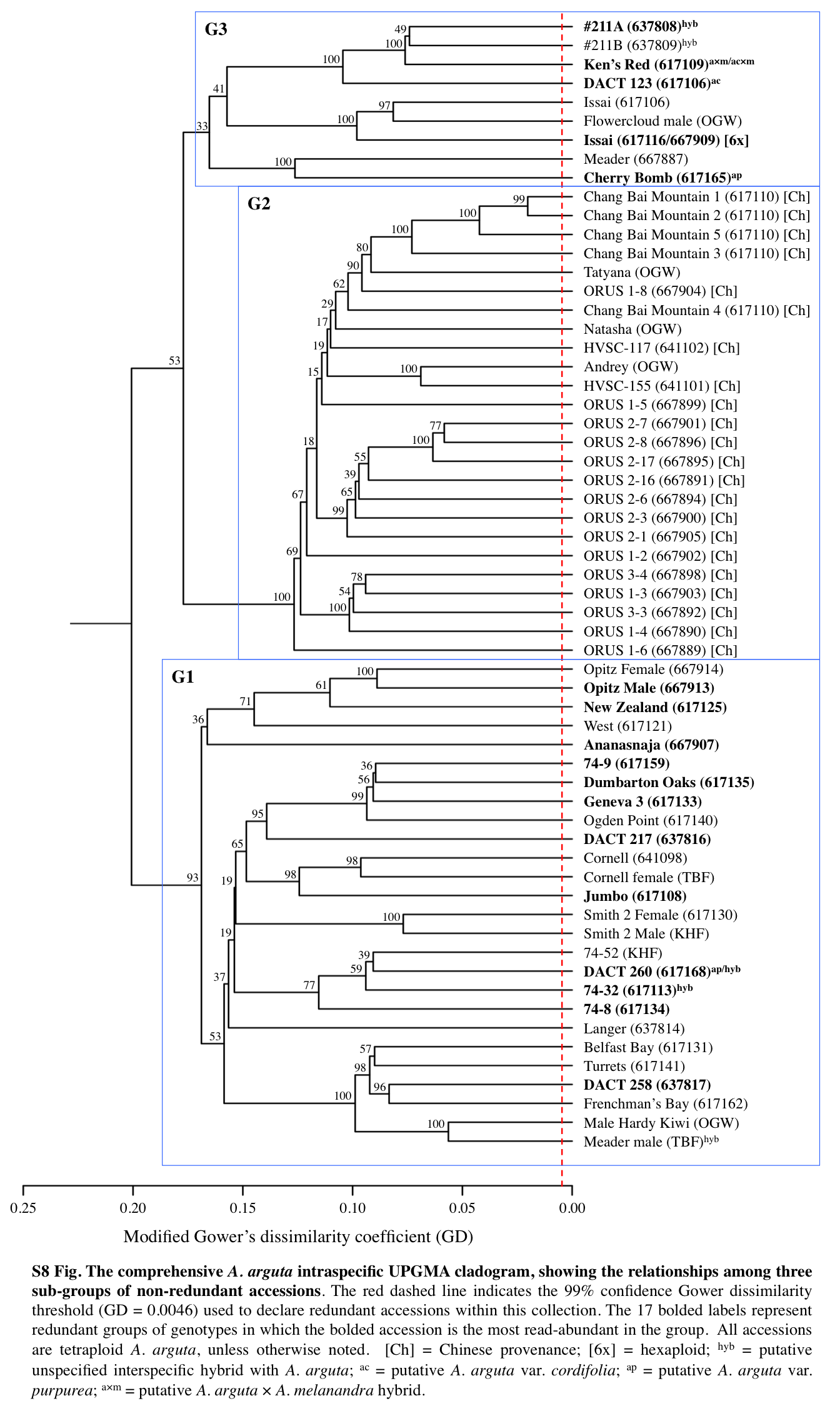

Supplement: S8 Fig — The red dashed line indicates the 99% confidence Gower dissimilarity threshold (GD = 0.0046) used to declare redundant accessions within this collection. The 17 bolded labels represent redundant groups of genotypes in which the bolded accession is the most read-abundant in the group. All accessions are tetraploid A. arguta, unless otherwise noted: [Ch] = Chinese provenance; [6x] = hexaploid; hyb = putative unspecified interspecific hybrid with A. arguta; ac = putative A. arguta var. cordifolia; ap = putative A. arguta var. purpurea; a×m = putative A. arguta × A. melanandra hybrid. (TIF) [file pone.0170580.s011.tif]

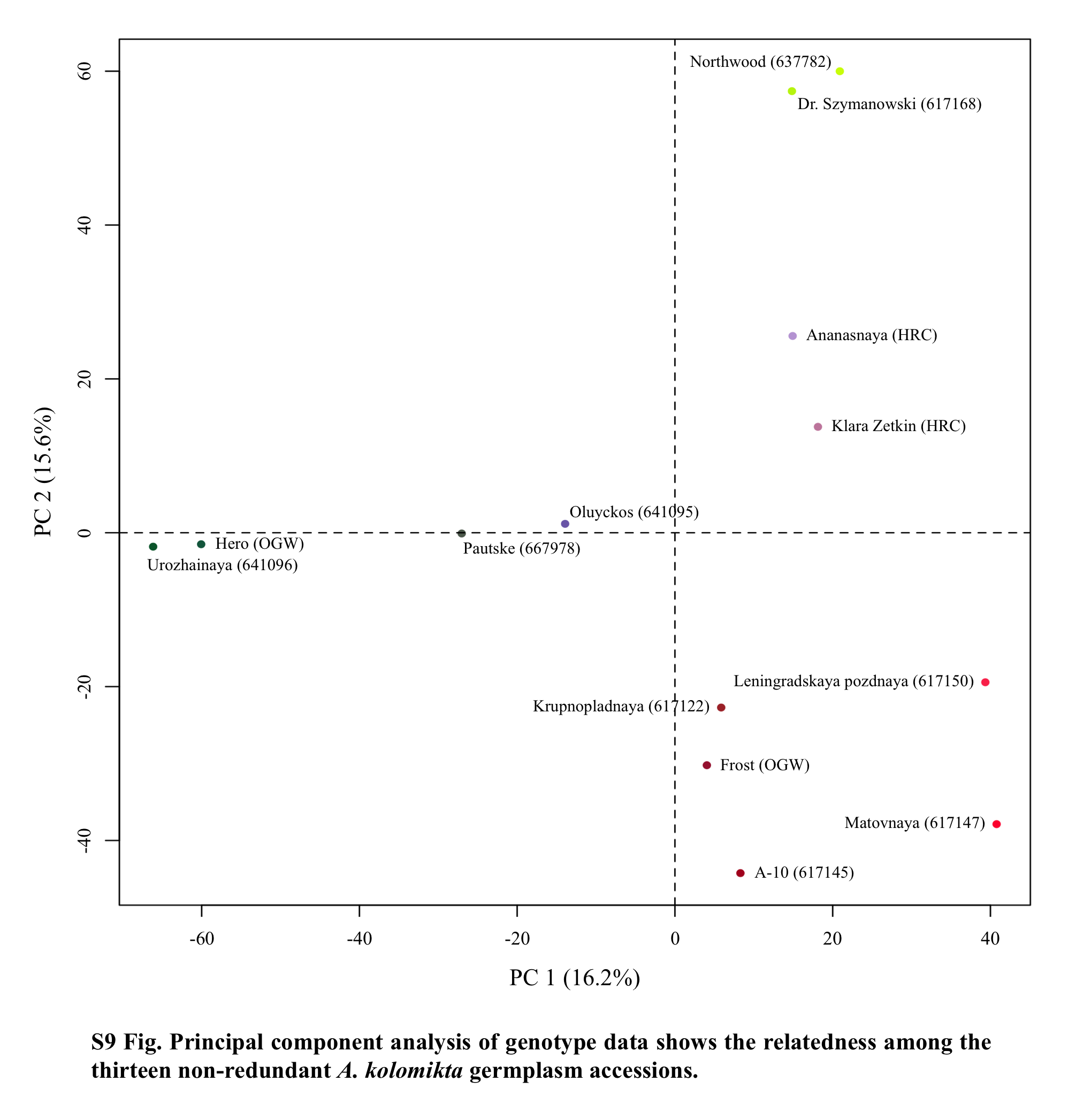

Supplement: S9 Fig — (TIF) [file pone.0170580.s012.tif]
